# Fire blight host-pathogen interaction: proteome profiles of *Erwinia amylovora* infecting apple rootstocks

**DOI:** 10.1038/s41598-018-30064-x

**Published:** 2018-08-03

**Authors:** Michelle Holtappels, Jean-Paul Noben, Patrick Van Dijck, Roland Valcke

**Affiliations:** 1 Molecular and Physical Plant Physiology, Faculty of Sciences, Hasselt University, Diepenbeek, B-3590 Belgium; 2 KU Leuven, Laboratory of Molecular Cell Biology VIB-KU Leuven, Center for Microbiology, Institute of Botany and Microbiology, KU Leuven, B-3001 Heverlee - Leuven, Belgium; 3 Department of Molecular Microbiology, VIB, B-3001 Heverlee - Leuven, Belgium; 4 School of Life Sciences, Biomedical Research Institute, Hasselt University and Transnational University Limburg, Hasselt, B-3590 Belgium

## Abstract

Fire blight, caused by the enterobacterium *Erwinia amylovora*, is a destructive disease, which can affect most members of the *Rosaceae* family. Since no significant genomic differences have been found by others to explain differences in virulence, we used here a gel-based proteomic approach to elucidate mechanisms and key players that allow the pathogen to survive, grow and multiply inside its host. Therefore, two strains with proven difference in virulence were grown under controlled conditions *in vitro* as well as *in planta* (infected apple rootstocks). Proteomic analysis including 2DE and mass spectrometry revealed that proteins involved in transcription regulation were more abundant in the *in planta* condition for both strains. In addition, genes involved in RNA processing were upregulated *in planta* for the highly virulent strain PFB5. Moreover, the upregulation of structural components of the F_0_F_1_-ATP synthase are major findings, giving important information on the infection strategy of this devastating pathogen. Overall, this research provides the first proteomic profile of *E*. *amylovora* during infection of apple rootstocks and insights into the response of the pathogen in interaction with its host.

## Introduction

The Gram-negative enterobacterium *Erwinia amylovora* is a plant pathogen that causes fire blight, a devastating necrotic disease forming a major threat to pome fruit and other economically relevant species belonging to the *Rosacea* family^1^. To date, no efficient and sustainable management strategy is available to fight fire blight. This is due to the limitations on the use of antibiotics, the occurrence of resistance against the existing antibiotics and the limited efficacy of the alternative control agents^2^.

The molecular basis of survival and propagation of virulent strains of *E*. *amylovora* inside its host is largely unknown and poorly understood. Much research has been performed on the host-pathogen interaction at the transcriptome level of the host^3–6^. Information on virulence factors and defense processes of the pathogen itself against the immune responses of the plant came from mutant screenings under controlled conditions, unable to completely mimic natural conditions. Several genes were shown to be upregulated upon infection of immature pear tissue such as virulence factors already known as components of the type III secretion system and the effector *dspE*^7^. This type III secretion system is encoded by genes from the *hrp* (hypersensitivity response and pathogenicity) gene cluster^8^ and consists of a pilus-like structure that will deliver effector proteins directly into the plant cells^9^. This system, together with amylovoran production, motility and biofilm formation is downregulated during infection by the secondary messenger cyclic di-GMP^10^.

Over the past decade, proteomic approaches have proven to be an excellent tool to characterize and understand the dynamic interplay of host and pathogen^11^. Therefore, this technique was chosen for investigating the proteome of *E*. *amylovora*. Moreover, due to its rather high conserved genome in comparison with other phytopathogenic bacteria, as published by *Mann et al*., where they did a comparative genomic analysis of 12 strains of *E*. *amylovora* and concluded that the chromosomes of *Spiraeoideae*-infecting strains are highly homogeneous^12,13^, we opted to look at the proteome of two strains to investigate the reactions the plant induces in the pathogen.

In previous studies of our lab, two-dimensional electrophoresis was successfully used to explore different aspects of the proteome of *E*. *amylovora*. We were able to identify differentially expressed proteins and genes between strains differing in virulence^14,15^. Moreover, we mapped the outer membrane proteome of *E*. *amylovora* grown *in planta* using a low and high virulent strain i.e. LMG2024 and PFB5, respectively^16^. We showed that the higher virulent strain produced more type III secretion effectors, which are necessary for starting and sustaining a successful infection^16^. Here, using an identical experimental setup, the focus was on the biochemical processes involved in a successful infection by comparing the CHAPS-urea solubilized protein complement by 2DE of the same two strains when grown *in planta*.

## Results

### Proteome analysis of *E*. *amylovora* during interaction with apple rootstocks

**Figure 1:**
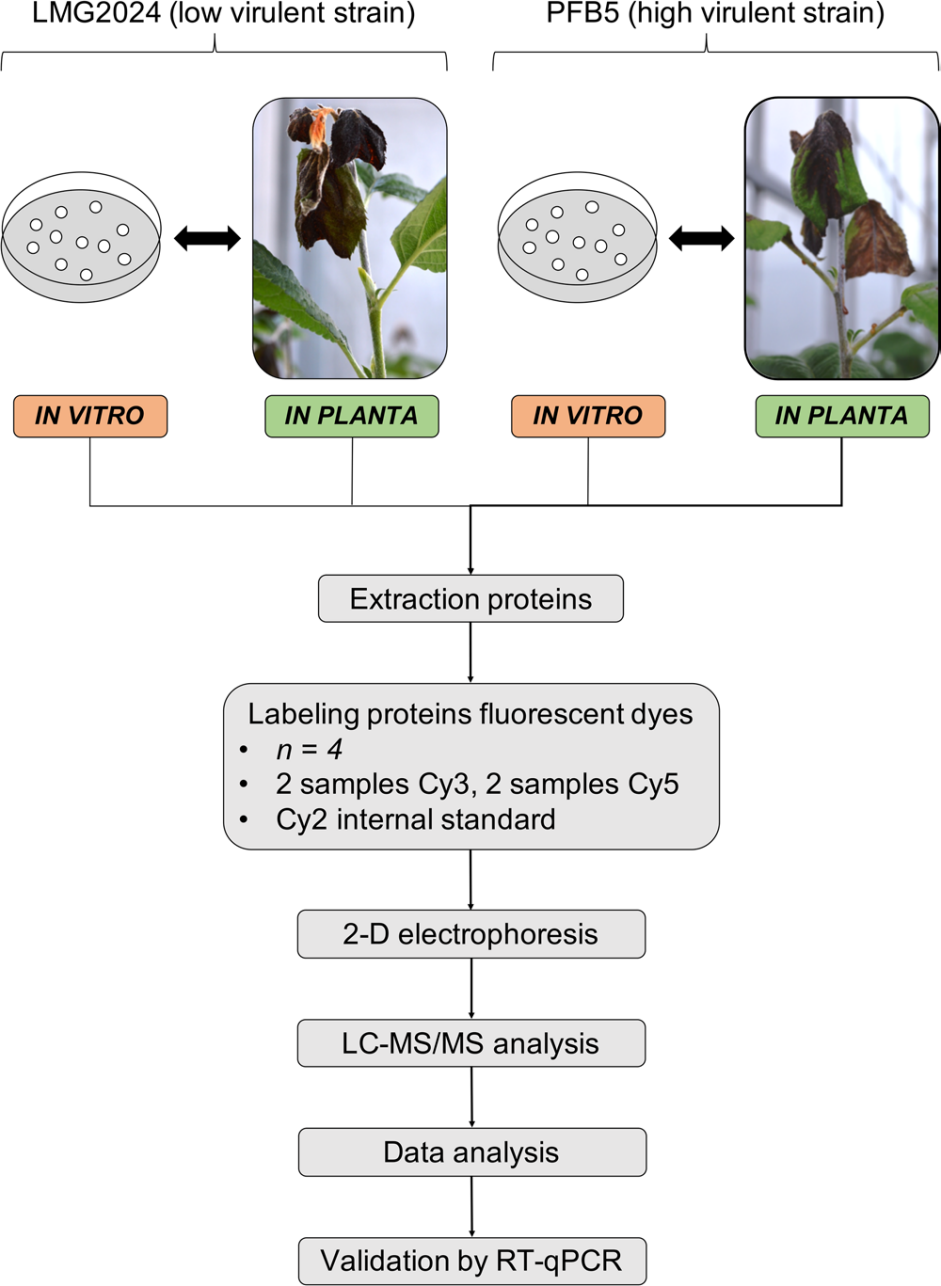
General workflow and experimental design of the proteomic experiment. Two strains differing in virulence were selected, LMG2024 (low virulent strain) and PFB5 (high virulent strain). Both strains were grown both *in vitro* and *in planta*. When grown until mid-exponential phase, the *in vitro* samples were taken. After 10 to 15 days after infection of apple rootstocks with respectively PFB5 and LMG2024, bacterial cells were extracted from the plant tissue. Samples from both strains and both conditions were used in a single 2D electrophoresis experiment so both strains and conditions could be compared using the SameSpot software. LC-MS/MS and data analysis was performed to identify the proteins. Finally, data were validated using RT-qPCR.

Cells were grown *in vitro* in MM_2_ medium until mid-exponential phase before protein extraction. For the *in planta* samples, apple rootstock were infected using the scissors method^15^ and when signs of systemic infection were observed, samples were taken, approximately after 10 to 15 days after infection depending on the strain^14^. The experimental set-up allowed a comparison between the *in vitro* and *in planta* proteome of both strains independently as well as a comparison between strains and conditions (Fig. 1). Thereby unraveling the mechanisms that could be pathogen specific but also highlighting differences between a high and low virulent strains.

### Comparison *in vitro* and *in planta* proteome of LMG2024

**Figure 2:**
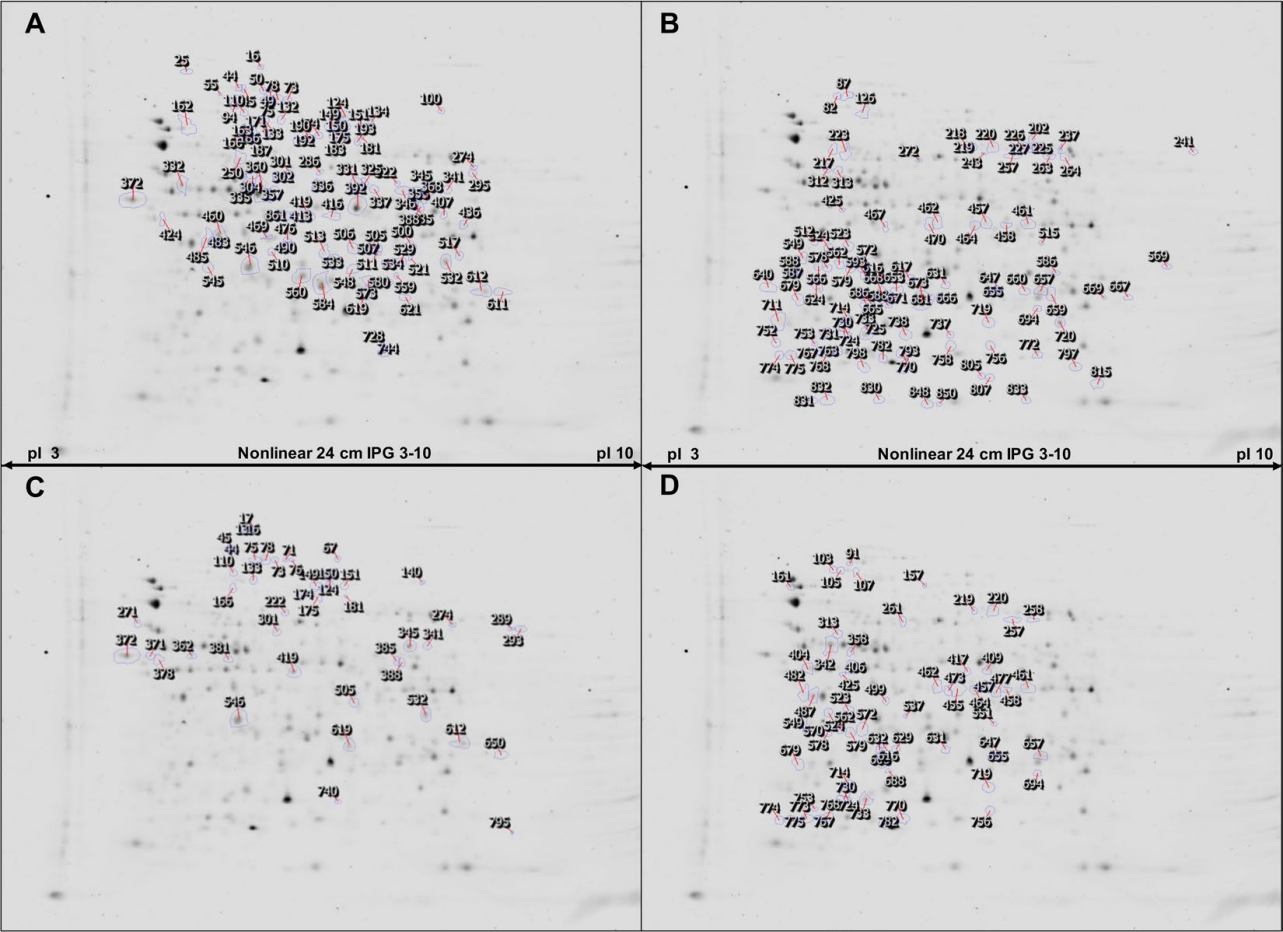
Representative 2DE gels of proteins extracted from *E*. *amylovora*. (**A**) Spots indicated represent proteins that are upregulated *in vitro* for LMG2024 in comparison with the *in planta* condition. (**B**) Spots upregulated *in planta* for LMG2024. (**C**) Indication of the spots upregulated *in vitro* in PFB5 in comparison with the *in planta* proteome (**D**). Upregulated spots from the *in planta* proteome in comparison with the *in vitro* proteome of PFB5.

**Figure 3:**
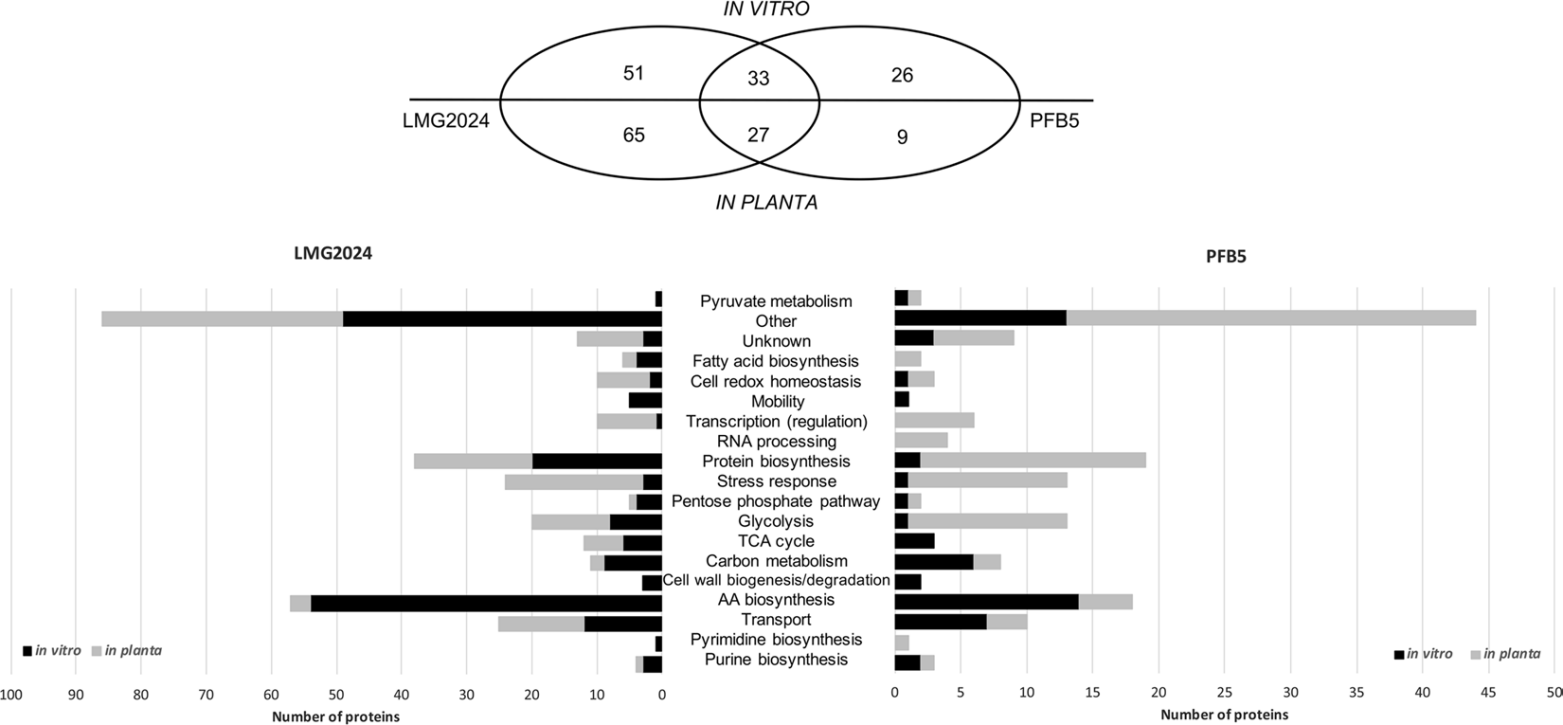
Functional categorization of the significantly different expressed proteins. Proteins identified in *E*. *amylovora* after 2DE are categorized according their biological and molecular function. This categorization was done for both strains and both conditions, *in vitro* represented in black and *in planta* in grey. The Venn diagram shows the proteins that were specifically and commonly identified between LMG2024 and PFB5 both *in vitro* and *in planta*.

When comparing the proteome *in vitro* and *in planta* of LMG2024, 200 spots were selected (Fig. 2A,B), meeting the preset requirements^14^. From these, 177 spots were identified by mass spectrometry, of which 93 were upregulated *in vitro* and 84 *in planta* (See Supplementary Data S1). For some spots, multiple proteins were identified having similar isoelectric point and molecular weight. Because of the uncertainty of which protein caused the difference between conditions, all proteins were considered. The identified proteins were further categorized according to their molecular function or biological process following the annotation of UniProtKB (www.uniprot.org) (Fig. 3). The percentage of each category was determined relative to the total amount of proteins that are identified. Moreover, if the same protein was identified in multiple spots, due to the uncertainty of possible isoforms of this protein, all were considered in the categorization. The main biological processes and molecular functions identified during this research include pyruvate metabolism, fatty acid biosynthesis, cell redox homeostasis, mobility, translation, transcription (regulation), protein biosynthesis, stress response, pentose phosphate pathway, glycolysis, TCA cycle, carbon metabolism, cell wall biogenesis/degradation, amino acid biosynthesis, transport, pyrimidine biosynthesis and purine biosynthesis.

It becomes clear that higher concentrations of flagellin are produced in a synthetic medium and this protein is less recovered *in planta*. Moreover, we see *in vitro* a higher amount of proteins involved in biosynthesis of amino acids while *in planta* more proteins involved in transcription and transcription regulation (RpoA, OmpR, EAMY_1829, GlnK, H-NS and CspG) are more abundant. Also, as expected a rise in proteins related to stress response are observed in higher amounts for the *in planta* condition.

### Comparison *in vitro* and *in planta* proteome of PFB5

The same comparison was made for a strain with a higher virulence, PFB5 (Fig. 2C,D). Out of 111 spots that were selected using the SameSpot software and that were picked from the gels, 62 were identified of which 46 were upregulated *in vitro* and 65 were upregulated *in planta* (See Supplementary Table S1). Also for this strain, the identified proteins were classified according their biological or molecular function (Fig. 3). *In vitro*, we saw an upregulation of proteins classified under the TCA cycle, cell wall biogenesis and degradation, amino acid biosynthesis and transport. Whilst for the *in planta* condition, proteins related to transcription (regulation), RNA processing, protein biosynthesis and stress response were more abundant.

### Corresponding results between both strains

**Figure 4:**
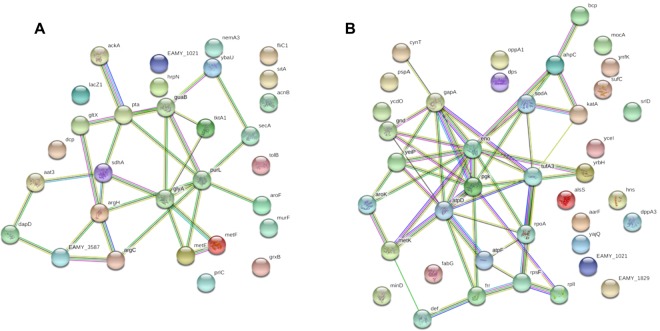
Interaction network of differentially expressed proteins. For both conditions, *in vitro* (**A**) and *in planta* (**B**) an interaction network was developed containing all proteins shared by both strains in this condition. Colored nodes represent query proteins and first shell of interactions. Empty nodes indicate proteins of unknown 3D structure. Different line colors indicate the types of protein-protein associations: light blue (from curated databases) and purple (experimentally determined) indicate known interactions. Green (gene neighborhood), red (gene fusions), blue (gene co-occurrence), light green (text mining), black (co-expression) and light purple (protein homology) indicate predicted interactions.

As can be expected, several proteins are shared amongst conditions between both strains. STRING was used to identify relationships between these proteins (Fig. 4, Supplementary Tables S3 and S4). This tool demonstrates the significance of the identified interactions. For the common proteins upregulated *in vitro* for LMG2024 and PFB5, the program identified 17 proteins as classified under metabolic pathways and 12 were classified as involved in biosynthesis of secondary metabolites. The other classifications included amino acid metabolism (9 proteins), microbial metabolism in diverse environments (9 proteins), carbon metabolism (7 proteins), propanoate metabolism (3 proteins), methane metabolism (3 proteins), taurine and hypotaurine metabolism (2 proteins), 2-oxocarboxylic acid metabolism (3 proteins), one carbon pool by folate (2 proteins), glyoxylate and dicarboxylate metabolism (2 proteins) and the biosynthesis of lysine (2 proteins).

However, for the *in planta* condition, no functional enrichment in the networks was detected. Although, from this network it becomes clear enolase is interconnected with many other proteins and forms a link between several different proteins.

### Validation of the proteomics results by reverse transcription PCR

**Figure 5:**
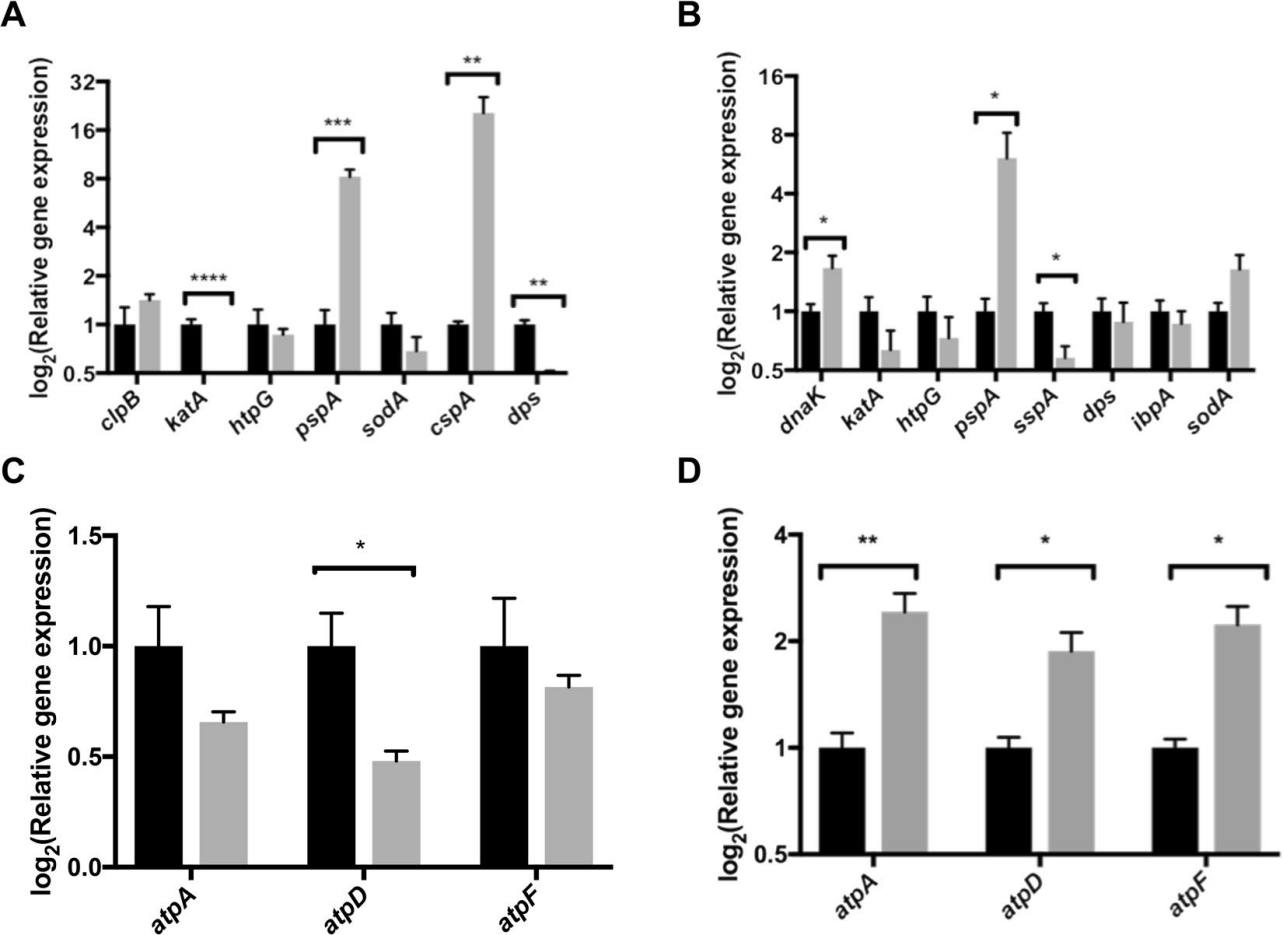
Gene expression profiling by RT-qPCR. Gene expression analysis was performed for genes involved in stress response for (**A**) LMG2024 and (**B**) PFB5 and (**C**) for genes corresponding to structural components of the F_0_F_1_-ATP synthase in the low virulent strain (LMG2024) and (**D**) the high virulent strain (PFB5). Normalized data are presented for the *in vitro* samples (black) and the *in planta* samples (grey). Up-or downregulations are presented on a log_2_ scale y-axis relative to the *in vitro* samples. Colums represent four biological replicates ± standard errors. Asterisk (*) indicates a statistically significance between conditions. The statistical analysis was done by unpaired student’s t-test, *p < 0,05; **p < 0,01; ***p < 0,001; ****p < 0,0001.

For *E*. *amylovora*, it is known that necrosis and the production of reactive oxygen species is induced by the pathogen through the injection of effector molecules and thereby aids in the infection process^17^. Therefore, we were especially interested in the stress related proteins that were identified during this screen. For the lower virulent strain, 6 stress related proteins were upregulated including ClpB, KatA, HtpG, PspA, SodA and CspG. For the higher virulent strain, we identified seven stress related genes including *dnaK*, *katA*, *htpG*, *pspA*, *sspA*, *dps* and *hspB*. For both strains, we used RT-qPCR to validate these results (Fig. 5A,B). Results show that for the lower virulent strain, proteomic and transcriptomic results correspond for PspA and CspG. However, for *katA* a contradictory result was obtained, showing that *katA* was lower expressed in the *in planta* condition in comparison with *in vitro* (Fig. 5A).

For the higher virulent strain, the data were confirmed for *dnaK* and *pspA*. For the other genes, no significant difference was observed between *in vitro* and *in planta* samples. However, for *sspA*, a contradictory result was observed (Fig. 5B).

Proteomic results also showed a higher abundance of structural proteins of the F_0_F_1_-ATP synthase including AtpA, AtpD and AtpF, which was observed for both strains. To validate these results, we measured the gene expression of the corresponding genes for both strains and both conditions. For the lower virulent strains, LMG2024, we found differences between proteomic and transcriptomic data (Fig. 5C). However, for PFB5, high correspondence with the proteomics data and significant differences could be observed for all three genes between both conditions (Fig. 5D).

## Discussion

The obtained results from this research show that these techniques can be used to extract viable cells from infected plant tissue for further processing using different omics techniques. For this study, a proteomics approach was chosen and interesting protemic data were validated using RT-qPCR.

From this research, we found that proteins related to transcription and transcription regulation are important for *E*. *amylovora* for survival and growth inside the host plant. For the low virulent strain, LMG2024, a higher expression of OmpR was observed *in planta*. This protein is part of the two-component signal transduction system (TCST) OmpR-EnvZ which is widely distributed and well-studied in γ-proteobacteria and which has shown to have a role in virulence, amylovoran biosynthesis and swarming motility in *E*. *amylovora*^18^. In this complex, the transmembrane sensor EnvZ, is activated in response to acidic pH conditions and changes in osmolarity and will next phosphorylate OmpR, which will regulate expression of OmpC and OmpF, two major outer-membrane porins^19^. This is confirmed by our previous research where we identified OmpF as being more abundantly expressed in the outer membrane of the lower virulent strain LMG2024 when grown *in planta*^16^. Moreover, OmpR is also involved in regulation of several cellular processes including gene expression of flagellar components and fatty acid transport^20^. During our research, we previously found that the lower virulent strain produces a higher amount of flagellin (FliC) than the higher virulent strain in an *in vitro* comparison^14^. This raised the hypothesis that the pathogen might use this regulator to lower the amount of flagellin to diminish recognition possibilities by the host, since flagellin can be recognized as a pathogen associated molecular pattern (PAMP)^21^. GlnK was also identified as more abundantly expressed during the *in planta* condition for LMG2024. This regulatory protein is differentially expressed in *Pseudomonas aeruginosa* under swarming conditions^22^. This could form a link between the proven higher swarming motility of this strain and more virulent strains^14^.

For both LMG2024 and PFB5, we identified the histon-like nucleoid structuring protein (H-NS), a DNA-binding protein, encoded by *hns* as being upregulated in the *in planta* condition. Functions of this protein are well known in *E*. *coli* and include organizing and compacting of the DNA but it is also important for the global regulation of the expression of approximately 5% of all genes in *E*. *coli* including proteins involved in transcription, translation and in production of components of the cell envelope which are needed for the adaptation to varying environments^23^. Moreover, research on *Shigella* spp. has shown that the ecological fitness of this pathogen is closely interwoven with the silencing of virulence genes by H-NS. Also, for this pathogen it is known that within the host, silencing mediated by H-NS orchestrates a precise and hierarchical expression of virulence genes that occur in response to environmental and host related cues^24–26^. These findings provide evidence for a possible role for H-NS in infection of *E*. *amylovora* since this protein is upregulated for both strain in the *in planta* condition and opens possibilities for further research.

As previous identified, several proteins related to RNA processing were found to be more abundant for the higher virulent strain in comparison with the lower one in an *in planta* comparison^15^. Interestingly, during this research, several of these proteins were identified in higher amounts for the *in planta* condition in PFB5 in comparison with *in vitro* including enolase and polynucleotide phosphorylase. Therefore, these proteins, indicating a higher RNA processing, may have a major implication on the difference between this low and higher virulent strain in their ability to survive and infect their host.

The design of the experiment allowed us to identity proteins that were corresponding between conditions and between both strains (Fig. 1). Thereby, we were able to extract the proteins that were shared between strains for both the *in vitro* comparison (Fig. 4A) and *in planta* comparison (Fig. 4B). *In vitro*, a strong enrichment of pathways could be found between the shared proteins. For the *in planta* condition, there was no enrichment in pathways, although, a strong interconnection exists between the proteins (Fig. 4B). Amongst these proteins, we found SrlD, a protein involved in the conversion of sorbitol, the main transport sugar alcohol in apple and pear^27^, to β-D-fructose-6P. It has been shown that sorbitol provides the pathogen with a good carbon source for the production of amylovoran and increases exopolysaccharide biosynthesis^28^ and that SrlD is required for the fire blight symptom formation on apple seedlings^29^. This protein can be of aid for the pathogen to overcome recognition by the immune system of the plant and help it to survive inside the host by allowing it to use sorbitol as a carbon source.

For this research, we were especially interested in the stress related defenses the pathogen uses to cope with the immune system of the plant. In their defense against pathogens, plants rely on the innate immunity of each cell and on systemic signals generated at the infection sites to fight off infection^30^. Upon infection with *E*. *amylovora*, an oxidative stress response is induced in the host including an accumulation of superoxide, lipid peroxidation, electrolyte leakage and enzyme induction, resembling an incompatible interaction^17^. Results show that several stress mechanisms are addressed for both strains. Similar proteins between both strains are observed of which PspA appears to be an important one. This protein is the major product of the *pspABCDE* operon in *E*. coli and in *Yersinia enterocolitica*, and plays an important role in the perception of membrane stress and consecutively signaling to the transcription apparatus, thereby using an ATP-hydrolysing transcription activator to produce effector proteins to overcome the stress^31^. Further investigation in *E*. *amylovora* would be necessary to prove that the same mechanism is present, however, this further emphasizes the importance of transcription regulation during the infection process. As previously reported, we see an upregulation of CspG, a cold shock protein for the lower virulent strain and an upregulation of DnaK, a heat shock protein, in PFB5^15^.

Remarkably, our proteome analysis showed a higher abundance of structural components of the F_0_F_1_-ATP synthase both for LMG2024 as PFB5. Transcriptome analysis only confirmed this for the higher virulent strain, PFB5 (Fig. 5C,D). Differences between proteome and transcriptome data may be observed in some cases^32^. Proteome data give information on the presence of a specific, final protein, whilst transcriptomics does not include posttranslational modifications and other processes that have an influence on the final protein product. Two important physiological functions have been assigned to the F_0_F_1_-ATP synthase in bacteria including oxidative phosphorylation by the synthesis of ATP aerobically from ADP and inorganic phosphate using the energy of an electrochemical ion gradient and the reverse reaction whereby a transmembrane ion gradient or proton motive force (PMF) is generated anaerobically at the expense of ATP hydrolysis when the driving force is low^33^. The generation of this PMF could be important to overcome an acidic environment by the increase of the intracellular pH^34^. It is known that pathogenic elicitors can induce a cytosolic acidification due to H^+^ influx in plants^35–37^. An upregulation of structural proteins of this F_0_F_1_-ATP synthase in *E*. *amylovora* during infection, may be important for acid tolerance induced by the plant as a defense mechanism. Moreover, both proteome as transcriptome results show a strong upregulation of these proteins/genes for the higher virulent strain PFB5 (Fig. 5D). Previously, we reported a higher gene expression of nearly all type III secreted effectors for this higher virulent strain^16^, which are possible inducers of cytoplasmic acidification by the plant. However, this hypothesis must be further investigated to prove the effective role of the F_0_F_1_-ATP synthase in acidic tolerance in *E*. *amylovora*.

In conclusion, this research highlights the importance of transcription and its regulation for the infection and survival strategy of *E*. *amylovora*. Moreover, a possible link was identified between stress perception at the membrane and effector production through PspA. Also, a higher expression of structural components of the F_0_F_1_-ATP synthase during infections indicates an effective mechanism against cytoplasmic acidification of the plant. Thereby these results form a firm platform for further investigation.

## Materials and Methods

### Bacterial strains and growth conditions

During this research, we included two strains of *E*. *amylovora* with a proven difference in virulence, namely LMG2024, a low virulent strain and PFB5, a high virulent strain^14–16^. For the *in vitro* experiments, the strains were grown overnight in liquid MM_2_ medium supplemented with 1% sorbitol^28^, shaking at 100 rpm at 24 °C. The bacteria were grown until the exponential phase (OD_600nm_ = 0.8).

For the *in planta* experiments, the bacteria were extracted from the infected apple rootstocks as described previously^15^.

### Protein extraction

The isolated bacteria from both *in vitro* and *in planta* experiments were washed in PBS; total protein fractions were extracted as described previously^14,15^. In short, after washing, the cells were disrupted by the addition of a lysis buffer (7 M urea, 2 M thio-urea and 4% (w/v) CHAPS) and sonication using a microtip. After a final centrifugation step (76 000 g for 90 min) the amount of proteins was determined using the 2-D Quant kit (GE Healthcare) according the instructions of the manufacturer.

### 2D DIGE

Next, analysis of the proteins was done using the differential in-gel electrophoresis (DIGE) for which samples were initially, minimally labeled using cyanine-derived fluors (3 Dyes 2D CYanine Labeling kit from Proteomics Consult) as described previously^14,15^. The individual Cy3, Cy5 and Cy2 labeled samples were mixed according to the experimental design including a dye swap. Each gel was loaded with 75 µg proteins, 25 µg from each sample and 25 µg from the internal standard. For this experiment, 4 biological replicates were considered for each strain and each condition. The separation in the first dimension was done with an IPGphor isoelectric focusing apparatus (GE Healthcare), using precast immobilized pH gradient (IPG) strips (SERVA; pH 3–10, 24 cm) on which the protein samples were loaded. The separation in second dimension was performed at 18 °C with an HPE-FlatTop Tower (SERVA) using precast, plastic-backed 10–15% polyacrylamide gels (2D-Large-Gel Flatbed NF 10–15% gradient gels) according to the manufacturer’s instructions. The fluorescent labeled proteins were visualized directly by scanning using an Ettan DIGE Imager (GE Healthcare). All gels were scanned at 100 μm (pixel size) resolution. Determination of spot abundance and statistical analysis were performed using the Progenesis SameSpot software. The spots considered, were spots with at least 1.5-fold changes in volume (P < 0.05) in one condition after normalization^14–16^. Following this analysis, the spots of interest were excised from the 2D gels in 1.5 mm diameter gel plugs using a semi-automated Screen picker (made by Proteomics Consult). Hereafter the plugs were processed for mass spectrometry according the protocol of Shevchenko *et al*.^38^.

### LC-MS/MS analysis and data analysis

The tryptic peptide mixture was further analyzed using an Easy-nLC 1000 liquid chromatograph (Thermo Scientific), on-line coupled to a mass calibrated LTQ-Orbitrap Velos Pro (Thermo Scientific) via a Nanospray Flex ion source (Thermo Scientific) using sleeved 30 µm ID stainless steel emitters (spray voltage +2.3 kV, capillary temperature: 200 °C) as described previously^14^. Next, the analysis of the mass spectrometric raw data was carried out using Proteome Discoverer software v.1.2 (Thermo Scientific) with build-in Sequest v.1.3.0339 and interfaced with an in-house Mascot v.2.4 server (Matrix Science). The Scaffold protein and peptide scoring and identification data obtained for each sample were exported and subsequently, merged into a Microsoft Excel summary report (Supplementary Data S1).

The UniprotKB database (www.uniprot.org) was used to annotate the identified proteins, using the GO sets ‘biological process’ and ‘molecular function’. Moreover, the proteins that were shared between both strains for each condition were presented in a graphical form using the Search Tool for Retrieval of Interacting Genes (STRING) (https://string-db.org/). This is a database and web resource dedicated to understanding the relationship among differentially expressed proteins. The protein interactions can be displayed according their confidence, evidence and actions or interactions.

### RNA extraction and gene expression analysis by quantitative RT-PCR

For the *in vitro* experiments, the *E*. *amylovora* strains were grown in liquid MM2 medium until the mid-exponential phase before adding 2 volumes of RNAprotect Bacteria Reagent (Qiagen, Venlo, The Netherlands). The *in planta* samples were treated with this reagent after the final wash step with PBS. Hereafter, the bacteria were collected by centrifugation (5000 g, 10 min) and the RNA was extracted using the RNeasy Mini Kit (Qiagen, Venlo, The Netherlands) as described previously^14^. Quantitative PCR (qPCR) was performed using Fast SYBR Green chemistry according to the manufacturer’s instructions on an ABI Prism 7500 Fast Real-Time PCR System (Applied Biosystems, Belgium). Relative gene expression was calculated as 2^−∆Cq^ and was normalized with a normalization factor determined using the GrayNorm algorithm according to Remans *et al*.^39^. For LMG2024, reference genes *proC* and *rpoS* were adequate whilst for PFB5 *rpsL* and *gyrA* provided the best outcome. Gene-specific primers based on proteins of interest indicated by the proteomics study were developed using Primer3 (Whitehead Institute/MIT Center for Genome Research) (Supplementary Table S2).

## Electronic supplementary material

Supplementary tables S2, S3 and S4

Supplementary data S1
