# Supplementary material for: Fire blight host-pathogen interaction: proteome profiles of Erwinia amylovora infecting apple rootstocks
Source: Sci Rep. 2018 Aug 3;8:11689. doi: 10.1038/s41598-018-30064-x (PMC6076297; doi:10.1038/s41598-018-30064-x)
Supplement: Supplementary file 1 — Supplementary tables S2, S3 and S4 [file 41598_2018_30064_MOESM1_ESM.pdf]

Supplementary tables

Fire blight host-pathogen interaction: proteome profiles of *Erwinia amylovora* infecting apple rootstocks

Michelle Holtappels[1,2,3], Jean-Paul Noben[4], Patrick Van Dijck[2,3] and Roland Valcke[1,*]

[1]Molecular and Physical Plant Physiology, Faculty of Sciences, Hasselt University, Diepenbeek, B-3590, Belgium

[2]KU Leuven, Laboratory of Molecular Cell Biology VIB-KU Leuven, Center for Microbiology, Institute of Botany and Microbiology, KU Leuven, B-3001 Heverlee - Leuven, Belgium

[3]Department of Molecular Microbiology, VIB, B-3001 Heverlee - Leuven, Belgium

[4]School of Life Sciences, Biomedical Research Institute, Hasselt University and Transnational University Limburg, Hasselt, B-3590, Belgium

*roland.valcke@uhasselt.be

Supplementary table S2.

Primers used in this study

Gene name	Forward Primer	Reverse Primer
Primers for reference genes LMG2024		
proC	GCCGGCCTATGTGTTTATGT	GATTGCGCTGCAAACTGATA
rpoS		
Primers for reference genes PFB5		
rpsL	AACTCCGCACTGCGTAAAGT	GGATCAGGATCACGGAGTGT
gyrA	CGAGCACGTTCATAGCGTAA	TATGCAATGTCGGTCATCGT
Primers for genes related to stress		
clpB	GCTTTACGCTGCATGTTTCA	GCTGCTGGATAGCTCGTTTC
katA	GCCAGTGATGAGCATTCCTT	TGCGTTCACGGTTGAAATTA
htpG	GGAAGGCCAGGAGAAGATTT	GAACCTCGATGCCTTTCTTG
pspA	AACAAAAGCTGACCGACCTG	CTTTCCAGCTCGCTGATTTC
sodA	CAGTTTGGCGATCAGCTCTT	CACACCAAACACCATCAAGC
cspA	AAGGTTTCGGCTTCATCTCC	TGACCTTCTTCCAGCGTCTT
dps	CGATATGCGCAAAGCTATCA	TGGACTCGATGAACCACAGA
dnaK	GTTAGTCCTGGCCATCGGTA	AGGGATTCAACTGCATCTGG
sspA	ATTGACCTCAACCCATACCG	GGAAAACGTTCGTCCAGGTA
ibpA	GGATAACCCCCGTTATTTCC	CGCTTTACCGTTCATCCATT
Primers for genes related to the F_0F_1-ATP synthase		
atpA	ACCGATTTGTAACCGGTCTG	GCTATCGACAACGACGGTTT
atpD	TATGCCTTCTGCGGTAGGTT	AGGTGATTGAACCGGTTTTG
atpF	TGGGTGACGATCTTGTTACG	CTCAGGTCATCATCGAGCAG

Supplementary table S3.

Proteins identified as higher abundant *in vitro* for both strains

Spot number	Description	Gene name	LMG2024		PFB5	
			Anova P-value	Fold change	Anova P-value	Fold change
16	phosphoribosylformylglycineamide synthetase	*purL*	*6,50E-04*	*8,5*	*0,002*	*4,6*
44	aconitate hydratase 2	*acnB*	*0,007*	*4,6*	*0,001*	*2,1*
45	aconitate hydratase 2<br>preprotein translocase subunit SecA	*acnB*<br>*secA*	*0,006*	*3,8*	*0,011*	*2,3*
73	5-methyltetrahydropteroyltriglutamate/homocysteine S-methyltransferase	*metE*	*7,95E-04*	*13,3*	*2,48E-04*	*6,8*
75	5-methyltetrahydropteroyltriglutamate/homocysteine S-methyltransferase	*metE*	*0,002*	*4,8*	*0,007*	*3,1*
78	5-methyltetrahydropteroyltriglutamate/homocysteine S-methyltransferase	*metE*	*4,92E-04*	*15,9*	*8,60E-05*	*9,5*
110	phosphate acetyltransferase	*pta*	*2,28E-04*	*3,2*	*0,014*	*2*
124	dipeptidyl carboxypeptidase II	*dcp*	*8,37E-06*	*2,4*	*0,003*	*4,1*
149	transketolase	*tktA1*	*4,04E-06*	*3,2*	*0,004*	*2,7*
150	transketolase	*tktA1*	*1,11E-05*	*11,7*	*3,65E-04*	*7*
151	transketolase	*tktA1*	*1,21E-06*	*16,6*	*2,92E-04*	*6*
166	Zn-dependent oligopeptidase	*prlC*	*0,002*	*2,9*	*0,003*	*2,3*
174	succinate dehydrogenase flavoprotein subunit	*sdhA*	*0,006*	*2,3*	*5,94E-04*	*2,5*
175	protease maturation protein	*ybaU*	*0,002*	*2,4*	*0,007*	*2,5*
274	beta-galactosidase/beta-glucuronidase<br>inosine-5'-monophosphate dehydrogenase	*lacZ1*<br>*guaB*	*0,012*	*2*	*0,011*	*2*
301	argininosuccinate lyase<br>glutamyl-tRNA synthetase	*argH*<br>*gltX*	*5,34E-05*	*4,8*	*0,005*	*2,2*
341	UDP-N-acetylmuramoylalanyl-D-glutamyl-2,6-diaminopimelate--D-alanyl-D-alanyl ligase<br>serine hydroxymethyltransferase	*murF*<br>*glyA*	*1,99E-05*	*11,6*	*0,007*	*3,6*
345	serine hydroxymethyltransferase	*glyA*	*1,31E-04*	*7,6*	*0,005*	*3,3*
372	harpin elicitor	*hrpN*	*0,005*	*4,8*	*0,003*	*10,2*

385	carboxypeptidase G2	*EAMY_3587*	5,52E-04	2,3	0,012	2,3
	acetate kinase	*ackA*				
	protein TolB	*tolB*				
	morphinone reductase	*nemA3*				
	bifunctional acetylornithine delta-aminotransferase/N-succinyldiaminopimelate aminotransferase	*aat3*				
388	acetate kinase	*ackA*	3,46E-04	3,9	0,001	2,9
	bifunctional acetylornithine delta-aminotransferase/N-succinyldiaminopimelate aminotransferase	*aat3*				
419	phospho-2-dehydro-3-deoxyheptonate aldolase	*aroF*	0,001	7	0,013	2,8
505	N-acetyl-gamma-glutamyl-phosphate reductase	*argC*	2,88E-05	10,9	2,05E-04	4,6
	5,10-methylenetetrahydrofolate reductase	*metF*				
532	iron ABC transporter substrate-binding protein	*sitA*	2,63E-05	11,1	1,20E-040	6,6
546	flagellin, filament structural protein FliC	*fliC1*	5,05E-05	10,1	0,007	2,5
	2,3,4,5-tetrahydropyridine-2,6-carboxylate N-succinyltransferase	*dapD*				
612	putative ABC transport system, periplasmic component	*EAIL5_0767*	9,80E-05	3,6	0,013	2,5
619	glutaredoxin-2	*grxB*	1,16E-06	4,6	0,012	2,6
	hypothetical protein EAMY_1021	*EAMY_1021*				

Supplementary table S4.

Proteins identified as higher abundant *in planta* for both strains

Spot number	Description	Gene name	LMG2024		PFB5	
			Anova P-value	Fold change	Anova P-value	Fold change
219	oligopeptide ABC transporter substrate-binding protein OppA	*oppA1*	*4,62E-04*	*6,7*	*0,009*	*3*
220	acetolactate synthase large subunit	*alsS*	*0,001*	*3,4*	*0,006*	*2,8*
	catalase	*katA*				
258	ABC transporter substrate-binding protein	*dppA3*	*0,001*	*7,9*	*0,013*	*2,9*
313	6-phosphogluconate dehydrogenase	*metK*	*0,01*	*2*	*0,014*	*3,3*
425	DNA-directed RNA polymerase subunit alpha	*eno*	*0,004*	*3,2*	*0,007*	*4,7*
	hypothetical protein EAMY_2128	*gnd*				
	phosphoglycerate kinase	*rpoA*				
457	transaldolase A	*ycdO*	*6,59E-04*	*3,1*	*8,13E-04*	*3,7*
	glyceraldehyde-3-phosphate dehydrogenase	*pgk*				
458	oxidoreductase	*mocA*	*7,46E-04*	*3,5*	*6,13E-04*	*3,1*
	glyceraldehyde 3-phosphate dehydrogenase A	*gapA*				
461	oxidoreductase	*mocA*	*3,96E-04*	*2,4*	*0,008*	*2,1*
	D-arabinose 5-phosphate isomerase	*yrbH*				
	glyceraldehyde 3-phosphate dehydrogenase A	*gapA*				
462	F0F1 ATP synthase subunit beta	*atpD*	*2,07E-04*	*4,1*	*0,002*	*5,3*
464	ABC transporter substrate-binding protein	*dppA3*	*2,79E-05*	*5,5*	*7,40E-05*	*4,3*
549	septum site-determining protein minD	*minD*	*0,001*	*2,5*	*0,004*	*2,2*
	FeS assembly ATPase SufC	*sufC*				
	enolase	*eno*				
570	septum site-determining protein minD	*minD*	*0,001*	*3,6*	*1,91E-04*	*3,5*
	elongation factor EF-P	*yeiP*				
	F0F1 ATP synthase subunit beta	*atpD*				
572	sorbitol-6-phosphate dehydrogenase	*srlD*	*0,002*	*2,3*	*0,005*	*3,7*
579	sorbitol-6-phosphate dehydrogenase	*srlD*	*3,06E-05*	*8,2*	*3,35E-06*	*4*
616	NAD(P)H-flavin reductase	*ubiB*	*1,57E-06*	*22,5*	*4,90E-04*	*6,3*
	hypothetical protein EAMY_1021	*EAMY_1021*				
	phage shock protein A	*pspA*				

631	dethiobiotin synthetase	*ynfK*	*0,003*	*3*	*0,008*	*2,7*
647	hypothetical protein EAM_0402	*EAM_0402*	*0,003*	*2*	*0,004*	*2,6*
655	hypothetical protein EAM_0402	*EAM_0402*	*9,22E-05*	*3,1*	*0,003*	*3,3*
	carbonate dehydratase	*cynT*				
657	3-oxoacyl-ACP reductase	*fabG*	*1,64E-05*	*4,4*	*0,01*	*2,3*
	superoxide dismutase, manganese	*sodA*				
668	alkyl hydroperoxide reductase subunit C	*ahpC*	*1,70E-04*	*5,3*	*0,002*	*4,7*
688	peptide deformylase	*def*	*0,015*	*3,6*	*0,002*	*2,5*
694	ribosome recycling factor	*frr*	*0,003*	*2,2*	*0,006*	*2,5*
	hypothetical protein EAMY_0970	*yajQ*				
719	hypothetical protein EAMY_1435	*yceI*	*0,007*	*5*	*0,007*	*3,6*
724	shikimate kinase I	*aroK*	*9,93E-05*	*6*	*3,45E-04*	*5,5*
	ATP synthase subunit B protein	*atpF*				
	DNA protection during starvation protein	*dps*				
730	DNA protection during starvation protein	*dps*	*0,003*	*4,1*	*0,002*	*4,3*
733	putative type VI secretion system, effector protein hcp	*EAIL5_3094*	*0,008*	*2,7*	*0,006*	*2,5*
	DNA protection during starvation protein	*dps*				
753	bacterioferritin comigratory protein	*bcp*	*0,001*	*13,2*	*0,008*	*8,5*
756	50S ribosomal protein L9	*rplI*	*0,009*	*3,2*	*0,011*	*2,4*
767	DNA-binding protein H-NS	*hns*	*0,007*	*3,7*	*0,001*	*4,1*
	30S ribosomal protein S6	*rpsF*				
768	DNA protection during starvation protein	*dps*	*7,45E-04*	*3,1*	*5,29E-04*	*3,2*
	global DNA-binding transcriptional dual regulator H-NS	*hns*				
	30S ribosomal protein S6	*rpsF*				
774	30S ribosomal protein S6	*rpsF*	*0,002*	*4,7*	*0,011*	*2,7*
775	30S ribosomal protein S6	*rpsF*	*0,01*	*3*	*0,003*	*2,9*
	hypothetical protein EAMY_1829	*EAMY_1829*				
